# Percutaneous ultrasound guided PEG-coated gold nanoparticles enhanced radiofrequency ablation in liver

**DOI:** 10.1038/s41598-020-79917-4

**Published:** 2021-01-14

**Authors:** Tudor Mocan, Rares Stiufiuc, Calin Popa, Iuliana Nenu, Cosmin Pestean, Andras Laszlo Nagy, Lavinia Patricia Mocan, Daniel Corneliu Leucuta, Nadim Al Hajjar, Zeno Sparchez

**Affiliations:** 1 3rd Medical Department, “Iuliu Hatieganu” University of Medicine and Pharmacy Cluj-Napoca, Cluj-Napoca, Romania; 2 Department of Bionanoscopy, MedFuture Research Center for Advanced Medicine, “Iuliu Hatieganu” University of Medicine and Pharmacy, Pasteur 4-6, 400337 Cluj-Napoca, Romania; 3 3rd Surgical Department, “Iuliu Hatieganu” University of Medicine and Pharmacy Cluj-Napoca, Cluj-Napoca, Romania; 4 Faculty of Veterinary Medicine, University of Agricultural Sciences and Veterinary Medicine, Calea Manastur no. 3-5, 400372 Cluj-Napoca, Romania; 5 Histology Department, “Iuliu Hatieganu” University of Medicine and Pharmacy Cluj-Napoca, Cluj-Napoca, Romania; 6 Medical Informatics and Biostatistics Department, “Iuliu Hatieganu” University of Medicine and Pharmacy, Cluj-Napoca, Romania

**Keywords:** Experimental models of disease, Nanoparticles, Hepatocytes

## Abstract

To investigate the effects of PEG-coated gold nanoparticles on ablation zone volumes following in vivo radiofrequency ablation of porcine liver. This prospective study was performed following institutional animal care and committee approval was used. Radiofrequency ablations were performed in the livers of ten Sus scrofa domesticus swines. During each ablation, 10 mL (mL) of Peg-coated gold nanoparticles at two different concentrations (0.5 mg/mL and 0.01 mg/mL) were injected through the electrode channel into the target zone. For the control group, 10 mL of physiological saline was used. Five to ten minutes after each ablation, contrast enhanced ultrasound (CEUS) was performed to evaluate the volume of the coagulation zone. On day five we performed another CEUS and the animals were sacrificed. Treated tissues were explanted for quantification of the ablation zones’ volumes. Hematoxylin and eosin (H&E) staining was also performed for histologic analysis. A total of 30 ablations were performed in the livers. The mean coagulation zone volume as measured by CEUS on day 5 after RFA was: 21.69 ± 3.39 cm^3^, 19.22 ± 5.77 cm^3^, and 8.80 ± 3.33 cm^3^ for N1, N2 and PS respectively. The coagulation zone volume after N1 and N2 treatments was significantly higher compared to PS treatment (p < 0.001 and p = 0.025 respectively). There was no difference between N1 and N2 treatment (p = 0.60). In our proof-of concept, pilot study we have shown for the first time that when injected directly into the target tissue during RFA, gold nanoparticles can substantially increase the coagulation zone.

## Introduction

In the last decades, radiofrequency ablation (RFA) has become an effective therapeutic method for both primary and secondary tumors. Over the time, it gained more and more popularity for physicians and patients, especially in the treatment of liver cancer. In patients with early hepatocellular carcinoma (HCC), RFA is used as a treatment option in non-surgical candidates^1^, and as a second-line treatment option (after surgical resection) in patients with colorectal cancer liver metastasis^2^. Although RFA has a couple of advantages over “classical” surgical resection (i.e. fewer complications due to a less invasive character) several issues are yet to be solved. Among them, the tumor size is probably the most important one. In general, RFA is only highly effective for tumors smaller than 3 cm (cm)^3^. Even for this scenario, the 5-years local tumor progression (LTP) rate can reach 27%^4^. In general, microscopic foci of cancer cells reside within 1 cm of the target tumor. However, in large nodules, they can reside further than 1 cm away as well^5^. We have recently shown that a margin size greater than 5 mm after percutaneous ablation provides better LTP-free-survival in patients with liver metastasis^6^. Others have shown that a margin size > 10 mm after tumor ablation is rarely associated with local recurrence in patients with colorectal cancer liver metastases^7^. Treatment of 30 mm liver cancer tumors can be curative but for better outcomes, a coagulation diameter of at least 35–40 mm is mandatory, a size difficult to reach by the current RFA devices. So it is clear that for tumors greater than 30 mm, RFA is less effective. In one study that used multivariate analysis, tumor size > 30 mm was found to be the only independent risk factor for incomplete ablation (*p* = 0.049)^8^. It was recently showed that overall survival for patients with incomplete ablation is lower compared to patients with complete ablation^9^. Under these circumstances, increasing the current 30 mm limit of RFA is an important medical need. For liver cancer nodules between 25 and 30 mm, an increase in tumor coagulation zone will ultimately decrease the risk of local recurrence, while in cases of medium size liver cancer (3–5 cm nodules) RFA might become a first line treatment modality.

In response to these clinical necessities, various new modalities have been developed and implemented in the field of tumor ablation. The following modalities have been used to increase to some extent the diameter of the ablated tumors: (a) several electrode insertions, however with increased morbidity^4,10^; (b) increasing the power and ablation time with the risk of damaging the surrounding structures; (c) using special electrodes (cluster type) or techniques (multipolar technique)^11,12^; (d) combining several techniques i.e. RFA + transartherial chemoembolization (TACE) for HCC > 3 cm^13^; (e) reducing tumor blood flow before heating and (f) increasing the thermal sensitivity of the tumor^14^.

This far, little attention has been devoted to developing concomitant agents capable of enhancing the transmission of radiofrequency energy within biological tissues. Among the envisaged solutions, nanoparticles have piqued the interest of the medical community for usage within thermal ablation. Among all classes of nanoparticles developed so far, gold nanoparticles are particularly attractive for several reasons: they are easily prepared^15^, binding of molecules to gold nanoparticles (GNP) is easily achieved^16,17^, no significant toxicity is reported and most importantly, GNP have already been tested in clinical trials^15,18,19^. One group from our university has developed a rapid, efficient and straightforward one-step synthesis of stable aqueous colloids of GNP coated with unmodified poly(ethylene)glycol (PEG) molecules (PEG-coated GNP)^16,17^. However, in vitro and in vivo application of PEG-GNP has not been tested. Gold, like most metals, is an excellent conductor of thermal energy.

Herein we describe the results of an in vivo ablation trial in swines with the hypothesis that PEG-coated GNP administration during RFA will increase coagulation volumes in liver.

## Results

### Macroscopic findings

**Figure 1:**
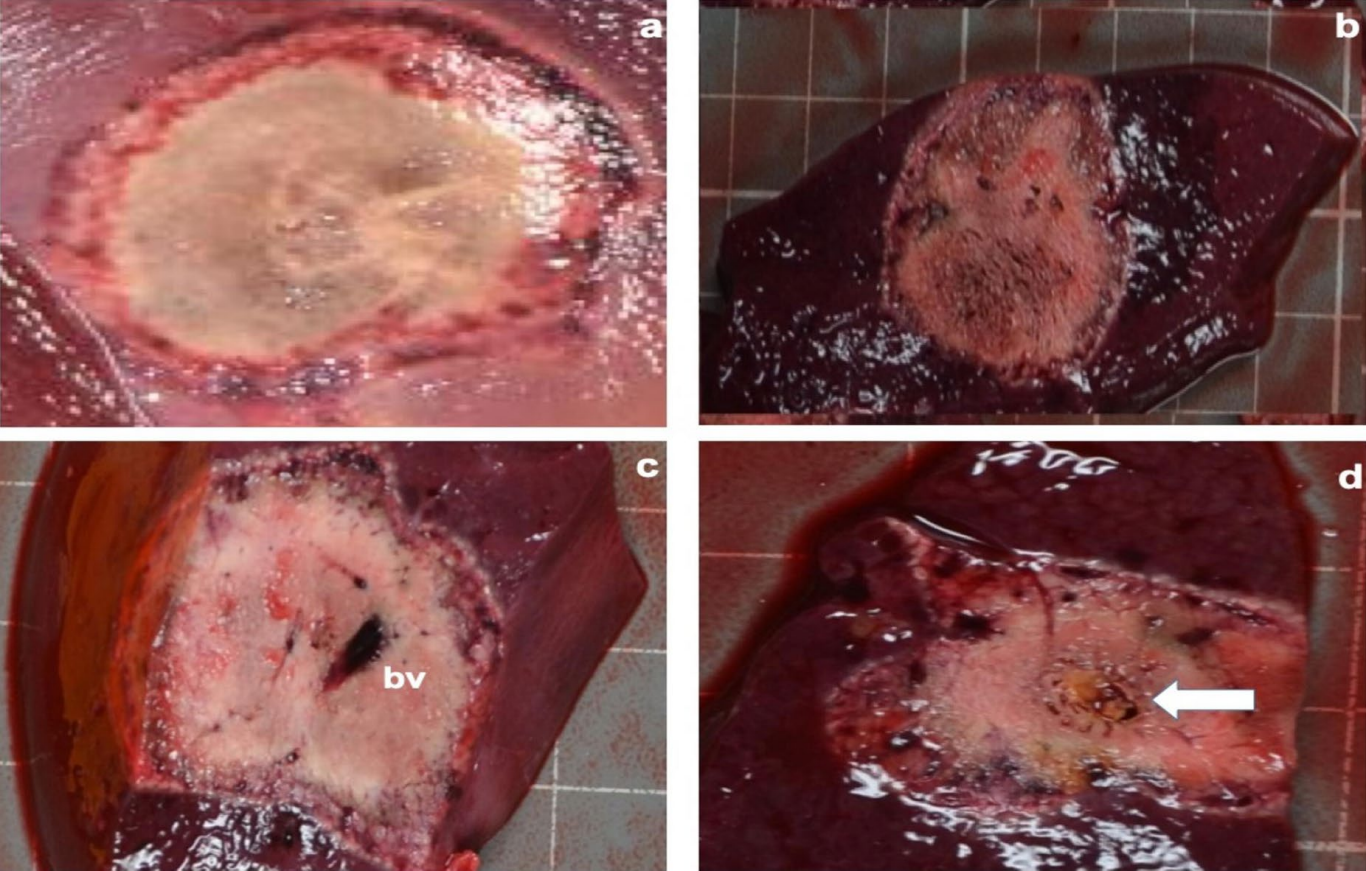
Macroscopic findings after RFA with or without PEG-coated GNP. The hemorrhagic rim (red zone) surrounding a central coagulation zone (white zone). (**a**) Photograph of the liver surface with the white and red zone. (**b**) Gross cross section of the components of the ablation zone. (**c**) There was no tissue charring when PEG-coated GNP RFA was used (bv = blood vessel). (**d**) Tissue charring in coagulation zone treated with physiological saline and RFA.

**Figure 2:**
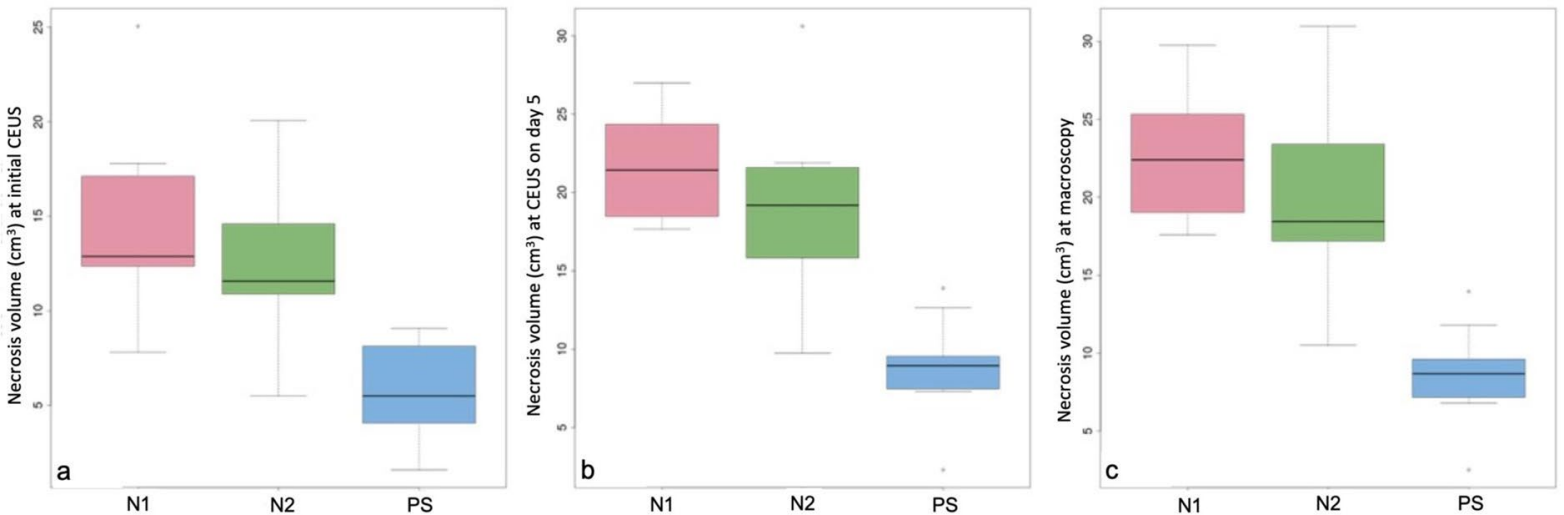
Coagulation zone volumes expressed as mean ± standard deviation of the three different treatments: N1, N2 and PS (physiological saline). (**a**) Coagulation zone volume measured with contrast enhanced ultrasound (CEUS) immediately after ablation. (**b**) Coagulation zone volume assessed with CEUS on day 5 after ablation. (**c**) Coagulation zone volume measured macroscopically with calipers.

A total of 30 ablations were performed in 10 pigs. Their mean weight was 29.92 ± 1.62 kg. After 5 days, the livers treated with RFA with or without PEG-coated GNP had ellipse-shaped thermal ablation zones. On cross sections of the ablation zones we could clearly identify a large central paleo-zone (the white zone) surrounded by a dark red rim (the red zone) (Fig. 1). Interestingly, there was little or no tissue charring in the lesions treated with PEG-coated GNP as opposed to those treated with physiological saline (PS) (Fig. 1). The mean coagulation zone volumes in the N1 (a solution containing 0.5 mg/mL of PEG-GNP), N2 (a solution containing 0.01 mg/mL of PEG-GNP) and PS group were 22.66 ± 3.95 cm^3^, 19.90 ± 5.55 cm^3^ and 8.63 ± 3.04 cm^3^, respectively. The difference was statistically significant between N1 and PS (p < 0.001), N2 and PS (p = 0.0045), but not between N1 and N2 (p = 0.40) (see Fig. 2 and Table 1 for further details).

**Table 1:** Macroscopic findings after RFA with or without gold nanoparticles.

Coagulation zone volumes									
Variable	N1 versus PS			N2 versus PS			N1 versus N2		
	N1	PS	p	N2	PS	p	p		
Mean in cm^3^ (SD)	22.66 (3.95)	8.63 (3.04)	< 0.001	19.9 (5.55)	8.63 (3.04)	0.004	0.40		
Median in cm^3^ (IQR)	22.4 (19.55–25.04)	8.69 (7.4–9.45)		18.43 (17.23–23.02)	8.69(7.4–9.45)				

Coagulation zone length, width, and height									
Variable	Length			Width			Height		
	N1	N2	PS	N1	N2	PS	N1	N2	PS
Mean in cm^3^(SD)	4.9 (0.57)	4.5(0.59)	3.4 (0.37)	2.9 (0.17)	2.8 (0.25)	2.1 (0.34)	2.9 (0.19)	2.8 (0.25)	2.1(0.32)
Median in cm^3^ (IQR)	5 (4.4–5.1)	4.6(4–4.9)	3.4 (3.3–3.5)	2.9 (2.8–3)	2.9 (2.8–2.9)	2.2 (2.1–2.3)	2.9(2.8–3.1)	2.8 (2.8–2.9)	2.19 (2–2.2)

*RFA* radiofrequency ablation, *SD* standard deviation, *IQR* interquartile range, *p* level of significance, *N1* ablation with the higher concentration of gold nanoparticles, *N2* ablation with the lower concentration of gold nanoparticles, *PS* physiological saline, *cm* centimeters.

### Microscopic findings

**Figure 3:**
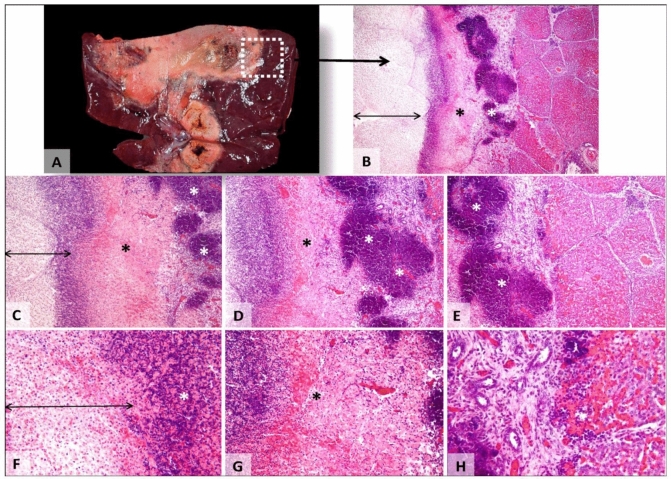
Gross and microscopic pictures of hepatic tissue after focal hyperthermia. (**A**) Massive necrosis focus involving all hepatic structures (white zone). (**B**–**H**) The red zone of thermic induced necrosis (double-headed arrow) and the adjacent, viable but highly congested hepatic tissue. The massive necrotic field involves all components of the hepatic tissue. It consists of coagulation necrosis, former cells presenting acidophilic and pale cytoplasm, nuclear pyknosis and karyorrhexis, while vessels display thrombi. Perilesional, there are a moderate post-necrotic congested and hemorrhagic border (black asterisk), massive, chronic and active inflammatory infiltrate (white asterisks) that tents to involve the coagulation zone and biliary hyperplasia. Towards the hepatic parenchyma, there is granulation tissue, with incipient fibroplasia and vasculogenesis. Hematoxylin and eosin stain, 40 × (**B**), 100 × (**C**), 200 × (**D**, **E**), 400 × (**F**, **G**, **H**).

Distinct histological regions of the thermal lesion were evident in H&E-stained sections of the RFA treated livers. These regions occurred concentric to the location of the RFA probe tract and were similar in shape and size to the macroscopically observed discolored lesions. The area immediately adjacent to the probe tract was mostly comprised of cell debris; bile ducts, vessels or parenchymal cells (the white zone) were completely absent. The dark red rim observed macroscopically (the red zone) corresponded to congestion, thrombosis and massive inflammatory infiltrate, with both chronic (lymphocytes) and acute (neutrophils) cells (Fig. 3). The pathologist classified the grade of inflammation for each red zone as either low, moderate or high. There was no difference between the degree of inflammatory cells infiltrates in the red zones treated by RFA versus red zones treated with RFA and PEG-coated GNP (p = 0.67). Moreover, the width of the red zone was not different (0.151 mm vs. 0.157 mm vs. 0.153 mm for N1 vs. N2 vs. PS respectively; p = 0.56).

### Ultrasound findings

**Figure 4:**
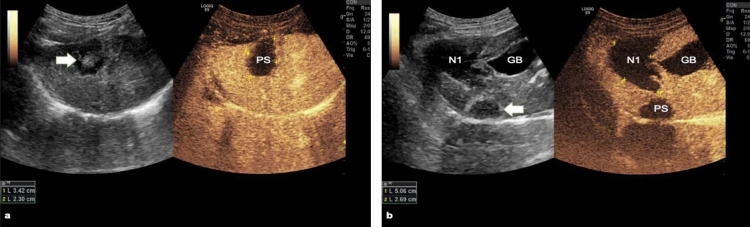
Ultrasound and contrast enhanced ultrasound evaluation of the coagulation zone. On conventional ultrasound, the coagulation zone could be clearly depicted (white arrows). (**a**) When physiological saline (PS) was used, a 3.4/2.3 cm coagulation zone was depicted at contrast enhanced ultrasound. (**b**) When PEG-coated GNP (N1) was used, the coagulation zone measured 5.09/2.69 cm. *GB* gallbladder.

**Figure 5:**
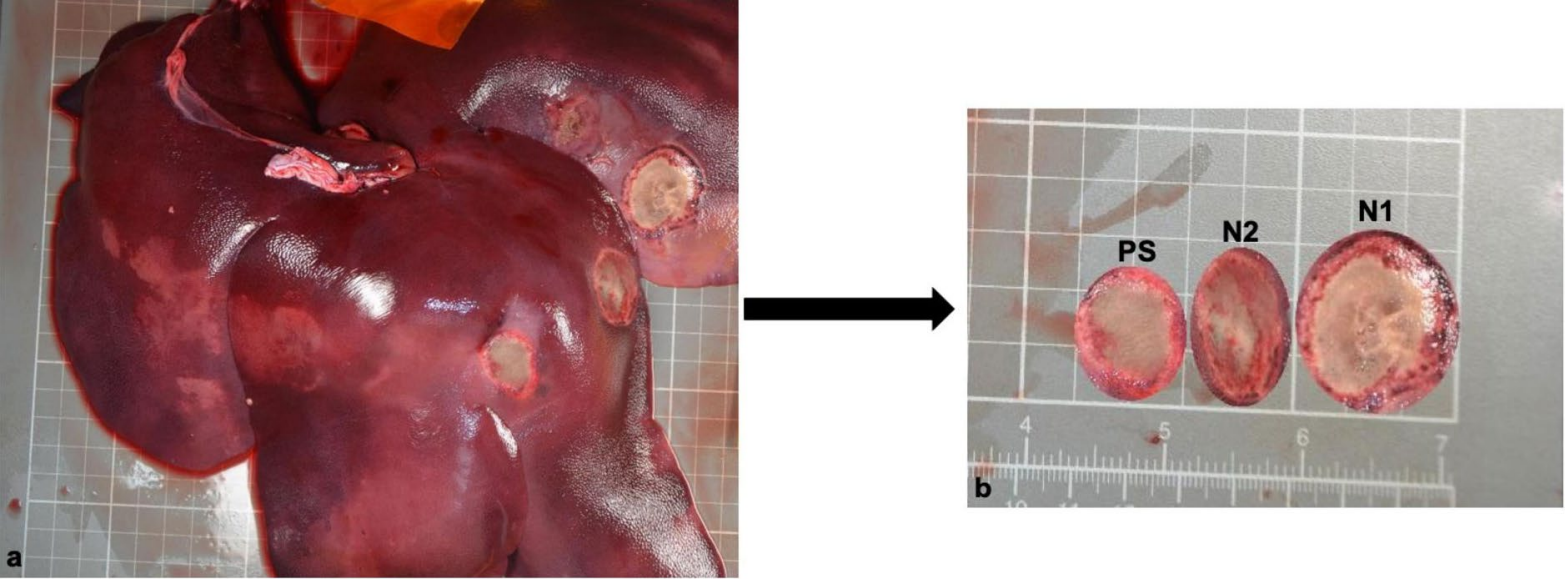
Ablation zones after RFA with physiological saline and PEG-coated GNP at two different concentrations. (**a**) Photograph of the liver surface showing three coagulation zones after RFA with or without PEG-coated GNP. (**b**) At a higher magnification, it can be clearly seen that PEG-coated GNP (N1) treated coagulation zone is almost two times bigger than physiological saline (PS) treated zone.

Conventional ultrasound and contrast enhanced ultrasound (CEUS) were performed 10 min after RFA and on day 5 after RFA. Using conventional ultrasound, the treated zones could be clearly visualized (Fig. 4), a finding which is different from clinical practice where it is often difficult to distinguish between treated and not treated zones. The mean coagulation zone as measured by CEUS immediately after RFA was: 14.32 ± 4.70 cm^3^, 12.53 ± 5.51 cm^3^ and 5.70 ± 2.61 cm^3^ for N1, N2, and PS respectively. The difference was statistically significant between N1 and PS (p = 0.014), N2 and PS (p = 0.013), but not between N1 and N2 (p = 0.545). The mean coagulation zone as measured by CEUS on day 5 after RFA was: 21.69 ± 3.39 cm^3^, 19.22 ± 5.77 cm^3^ and 8.80 ± 3.33 cm^3^ for N1, N2 and PS respectively. The coagulation zone after N1 treatment was significantly higher compared to PS treatment (p < 0.001) (Fig. 5 and Fig. 4). The coagulation zone after N2 treatment was significantly higher compared to PS treatment (p = 0.025). There was no difference between N1 and N2 treatment (p = 0.60) (Table 2 for more details). When comparing the mean coagulation zone volumes evaluated with CEUS performed immediately after RFA with those measured by CEUS 5 days after RFA, there was a statistically significant increase in volumes (p < 0.001 for N1, p < 0.001 for N2, and p = 0.009 for PS).

**Table 2:** CEUS results immediately after and on day 5 after RFA with or without gold nanoparticles.

Variable	N1 versus PS			N2 versus PS			N1 versus N2
	N1	PS	p	N2	PS	p	p
**Coagulation zone volumes as evaluated by CEUS immediately after treatment**							
Mean in cm^3^ (SD)	14.32 (4.7)	5.7 (2.61)	0.014	12.53 (4.35)	5.7 (2.61)	0.013	0.545
Median in cm^3^ (IQR)	12.86 (12.41–16.23)	5.5 (4.07–8.1)		11.57 (10.96–14.03)	5.5 (4.07–8.1)		
**Coagulation zone volumes as evaluated by CEUS on day 5 after treatment**							
Mean in cm^3^ (SD)	21.69 (3.39)	8.8 (3.33)	< 0.001	19.22 (5.77)	8.8 (3.33)	0.008	0.60
Median in cm^3^ (IQR)	21.43 (18.47–24.36)	8.95 (7.48–9.56)		19.18 (15.81–21.59)	8.95 (7.48–9.56)		

*CEUS* contrast enhanced ultrasound, *RFA* radiofrequency ablation, *SD* standard deviation, *IQR* interquartile range, *p* level of significance, *N1* ablation with the higher concentration of gold nanoparticles, *N2* ablation with the lower concentration of gold nanoparticles, *PS* physiological saline, *cm* centimeters.

### Complications

One major complication occurred in our study. One pig died five hours after treatment. Necropsy identified a large ventricular septal defect but no modifications in the abdomen. In this case we considered the cardiac malformation to be responsible for the death. In two other pigs, small diaphragmatic burns (one after N1 enhanced RFA and one after N2 mediated RFA) were seen on gross examination at the time of necropsy, while self-limiting peritoneal bleeding was present on necropsy in two other animals.

## Discussion

Improving RFA outcomes is an important medical need nowadays. Developing concomitant agents capable of enhancing the transmission of radiofrequency energy within biological tissues has become an appealing research field. Among concomitant agents, gold nanoparticles are particularly interesting. Like most metals, gold is an excellent conductor of thermal energy. More importantly, several gold nanoparticles are already used in clinical practice. Clinical trials with GNP in patients with prostate cancer have already shown promising results and a safe profile^19^. Nevertheless, several studies have shown that, when exposed to external RF fields, GNP injected intravenously accumulates into the tumors and thermally destroys them^20,21^. Others injected gold nanoparticles directly into the tumor and then exposed them to an external RF field, showing an increase in tumor coagulation zone^22^. However, no studies have injected GNP into the target zone during RFA. Thermal ablations were performed using a commercially available saline perfused 9 hooks RF electrode. Sodium chloride is well known as a concomitant agent for thermal ablation in clinical practice. For this reason, it was used as control in our study.

Using an in vivo swine model, we have herein demonstrated that the use of PEG-coated GNP as a concomitant agent during RFA in liver tissue significantly increased the coagulation zone volume. Overall we observed an increase of at least 0.5–1 cm in all axes (length, width, height) with the use of PEG-coated GNP. This is of particular importance since an ablative margin of 5 mm lowers the risk of local recurrences^6^, while a 1 cm ablative margin almost abolishes the risk of local recurrences^7^.

Possible mechanisms responsible for the coagulation zone augmentation are discussed below. One study has shown that the temperature increases directly with the GNP concentration up to a specific range and eventually saturates. Further increases in concentration had no effect on temperature changes^23^. In our study, we found similar coagulation volumes between N1 and N2. The increase in coagulation volumes was not dose-dependent. It is therefore possible that the concentration of GNP in N2 to be the maximum one and further increase in GNP concentration (e.g. N1) might not be able to produce further temperature changes. In this respect, further studies trying to identify the lowest concentration of GNP that can produce similar coagulation volumes to N1 and N2 are necessary. The size of GNP is also an important matter to be considered. Moran et al., showed that GNP with a diameter smaller than 50 nm have a twofold greater heating capacity compared to GNP with a diameter greater than 50 nm^24^. Similar to this, in our in vivo study we have confirmed that GNP (with a diameter of ~ 30 nm) increases the coagulation zone volume when exposed to RFA. We did not evaluate the mechanisms responsible for an increase in coagulation zone volume with the use of PEG-coated GNP. However, one group has demonstrated that GNP heat primarily via the Joule heating mechanism^25^. Furthermore, one study used carbon-coated iron nanofluid and reported similar results. Using an in vitro design, carbon-coated iron nanofluid was injected through the electrode channel in the swine liver during RFA. The target tissue temperatures were lower when carbon-coated iron nanofluid was injected (less than 100 °C) compared to the target tissue temperatures where PS was injected (higher than 100 °C) and there was no tissue charring around^26^. In our study, due to the in-vivo design it would be very difficult to place several electrodes at different distances around the electrode to monitor the target tissue temperatures. However, we did notice less or no tissue charring in zones treated with PEG-coated GNP. Therefore, we can suppose the same mechanism to be responsible for our results.

Many of our microscopic findings were similar to those already reported^22^. One study showed that the volume of the red zone was significantly smaller in tumors treated with tumor necrosis factor alpha-coated Gold Nanoparticles and RFA compared to RFA alone^13^. In our study, the width of the red zone was not different in N1 or N2 compared to PS treatment. Therefore, the increase in ablation volumes is secondary to an increase in coagulation zones and not to an increase or decrease of the red zones. Also important, we found no differences regarding the immune cells infiltrates in the treatment zones. Actually, it is well known that GNP (with a diameter < 50 nm) do not pose any risk of cellular injury or inflammatory responses^18^.

We decided to use CEUS to compare the coagulation zone volumes at different time intervals. CEUS is a well-established clinical tool to monitor the response to thermal ablation^27^. We did notice an increase in coagulation zone volumes over time (day 0 compared to day 5). However, the magnitude of the coagulation zone volume increase was similar in all treated zones (around 51% increases from baseline). Therefore, the PEG-coated GNP treated zones did not change significantly compared to PS treated tissue. In clinical practice, one study also reported an increase in the diameter of coagulation zone over time^28^.

We only encountered one major complication (death) during the 5 day follow-up, which was not related to GNP treatment. Only one other study with a similar design has been reported so far. However, they used a different concomitant agent (triphenyl tetrazolium chloride), microwave ablation (MWA), and they injected the concomitant agent before MWA. They have also showed that there was an increase in ablation volumes with the use of concomitant agents^29^. Moreover, they have defined three distinct zones: a zone of complete desiccation named zone 1; a zone of nonviable tissue but with retained lobular architecture named zone 2; and a zone of thin rim of congestion named zone 3. The increase was due to an increase of the zone 2 volume, suggesting that the use of concomitant agents facilitated microwave transmission. In our study, we could not delineate between zone 1 and zone 2 and we described a white zone (corresponding to zone 1 + zone 2) and red zone (corresponding to zone 3). We have shown that the increase in coagulation zone was due to increase in white zone, suggesting that when exposed to RF energy, PEG-coated GNP absorbs heat and then transfers it to surrounding structures. The capacity of GNP to absorb heat and then transfer it to surrounding structures has also been elegantly demonstrated by others. In a rat hepatoma model, intratumoral gold nanoparticle injections and then exposure to an external RF generator resulted in significant temperature increases and thermal injury as compared to vehicle (water) injected controls^30^. Nevertheless, it has been recently shown that GNPs are excellent RF responsive nonmaterial^31^.

We believe our approach is very close to clinical practice: (a) we used percutaneous ultrasound guided RFA in a swine model under profound sedation; (b) we injected PEG-coated GNP during RFA which is similar to clinical routine; (c) we used CEUS for the evaluation of response to treatment; (d) we closely monitored “patients” after RFA.

Our study has several limits. First, further ex-vivo studies measuring the temperature with and without GNP at various distances from the RFA electrode to identify the mechanism responsible for the increase in coagulation volumes observed here are necessary. Second, we used pigs with a median weight of 29.75 kg and we did encounter diaphragmatic burns (n = 2) and peritoneal bleeding (n = 2). Therefore, for these cases the measurement of coagulation volumes might be underestimated for some locations. However, this finding is less relevant as the differences between GNP ablations and saline ablations were clear in all cases. In further thermal ablation studies, animals weighting at least 70 kg should be used for more accurate results. And third, we did not evaluate whether, after RFA, GNP remain localized around the coagulation zone or migrate in distant tissues. However, PEGylated nanoparticles generally accumulate in the liver a half to a third of the amount of non-PEGylated NPs and demonstrate higher tumor accumulation versus background^32^. Moreover, PEG is inexpensive, versatile and FDA approved for many applications^33^.

In conclusion, our proof-of concept pilot study has shown for the first time that when injected directly into the target tissue during RFA, gold nanoparticles can substantially increase the coagulation zone.

## Methods

We estimated the sample size knowing that the coagulation zone after RFA in long axis is around three cm^27^. We set a minimum clinically interesting difference of one cm to be detected by the test comparing the short axis of the coagulation zone between PS-RFA and PEG-coated RFA. For the calculations, we used a power of 80% to detect the difference, and a two-tailed p level. The simulated sample size value obtained with these constrains was ten ablation sessions for each treatment type (G*Power 3.0.10 software, Universitat Dusseldorf, Dusseldorf Germany). Therefore, 30 ablation sessions in ten female Sus scrofa domesticus swines (mean weight, 29.2 kg) were performed. The study design and animal usage parameters described herein were reviewed and approved by the University of Agricultural Sciences and Veterinary Medicine from Cluj-Napoca, Romania (approval number: 241A/17/09/2015), and all husbandry and experimental studies were compliant with the National Research Council’s Guide for the Care and Use of Laboratory Animals. The study was conducted according to the ARRIVE guidelines for reporting animal research^34^.

### PEG-coated GNP

The PEG-coated GNP employed in this study have been synthesized by one group from our university. Complete details about synthesis^16^, cytotoxicity and physical properties^17^ of the PEG-coated GNP were already published. Twelve mL of a solution containing PEG-coated GNP were offered as a gift from Department of Pharmaceutical Physics-Biophysics, "Iuliu Hațieganu" University of Medicine and Pharmacy, Cluj-Napoca, Romania. The concentration of the GNP (according to nanoparticle tracking analyzer) was on the order of 5 × 10^11^ GNP/mL. For this study, two solutions containing PEG-coated GNP have been prepared and tested. The first one, (N1) has been prepared by mixing 10 mL of PEG-coated GNP with 90 mL of physiological saline (a solution containing 0.5 mg/mL of PEG-GNP) and the second one, (N2) has been prepared by mixing 2 mL of PEG-coated GNP with 98 mL of physiological saline (a solution containing 0.01 mg/mL of PEG-GNP).

### Study protocol

The ability of PEG-coated GNP to increase ablation zone volumes was evaluated by conducting an in vivo trial using 27–33 kg Sus scrofa domesticus swine females. We decided to use pigs because of a lack of large animal liver tumor model and because these livers are of sufficient size to allow the use RFA generators and electrodes similar to the ones used in clinical practice. For each pig, a total of three ablation sessions was performed (one session with PS perfused RFA, one session with N1 perfused RFA and one session with N2 perfused RFA), in different lobes. Each ablation session was performed at a distance of three cm from the other. The time between sessions was 30 min. The type of concomitant agent used for each session (PS, N1 or N2) was randomly decided by one investigator (C.P.). The investigators performing RFA (Z.S. and T.M) were not informed about the type of solution injected through the catheter during ablation. All ablations were performed with a 200 W generator (model 1500X; Rita Medical Systems, AngioDynamics) that was coupled to an expandable array with nine electrode tines. The electrode was placed into the target zone and deployed to 3 cm. The target zone was defined as a zone far from the gallbladder or major vessels (at least 2 cm far from major vessels). When reaching the target temperature (105 °C), the electrode tip was continuously perfused with physiological saline (PS) or PEG-coated GNP (N1 or N2) injected through the internal channel of the electrode until the end of the ablation session. At the end of the session, the generator was reactivated and the electrode track was ablated. Each session lasted 6 min. All ablation sessions were performed in the surgical department under profound analgosedation (by a board-certified doctor of veterinary medicine) using ultrasound guidance (Logiq E9, GE).

### Post-ablation monitoring

Ten minutes after each ablation session, contrast-enhanced ultrasound (CEUS) with SonoVue (Bracco, Italy) was performed to evaluate the coagulation zones in 3 axes. One mL of SonoVue was used for the measurement of one ablation session. SonoVue is an intravascular contrast agent that can easily delineate between vascular and avascular zones. After the injection of SonoVue, the ablation zone appeared as a dark, completely avascularized zone. The animals were kept alive for five days in separate rooms at a temperature of 22 degrees Celsius with access to food and water. During this period the animals were checked daily for signs of malaises, poor appetite, low activity level, respiratory difficulty or other signs of systemic illness. On day five, we performed another CEUS examination and evaluated the coagulation zone volumes. All measurements were stored for further analysis. Our intents were to see whether on day five after treatments, there was a change in terms of coagulation volumes. After CEUS examinations, all animals were euthanized (pentobarbital, > 88 mg/kg IV).

### Post-sacrifice analysis

Each ablation zone plus a two cm margin outside the ablation zone was visually identified and dissected out as a whole. All the ablation zones were cut in two axes: (a) perpendicular to the probe track for the measurement of the maximum length (L) and width (W); (b) parallel to the probe track for the measurement of the maximum height (H). The measurements of L, W and H were done with electronic calipers by T.M, who was blinded to the treatment used in each zone. We defined two different zone: a white zone of coagulative tissue and a red zone of hyperemia according to the guidelines of image guided tumor ablation reporting criteria^31^. Further on in the text, the term coagulation zone volume refers to white zone volume. Coagulation zone volumes were estimated with the equation of an ellipsoid volume = Pi/6*LWH. All probes were formalin (10%) fixed and paraffin-embedded. The ablation zones were further on cut into 4-µm thick slices perpendicular to the probe track using a Leica RM 2125 RT microtome. The tissue sections underwent standard histological processing with H&E staining. The slides were scanned at a magnification of 40X using an Olympus scan scope BX 41 scanner. The width of the red zone was measured using a digital Olympus UC 30 camera and the acquisition software Olympus stream basic. The widths of the red zones were calculated in the largest three different zones and summed to achieve the total width. A single pathologist with significant experience in thermal injury histology reviewed all slides and was blinded to the treatment groups.

### Statistical analysis

Continuous data were presented as means and standard deviations, as well as medians and interquartile ranges, due to the difficulty of assessing the normality of the data for small samples. Multiple comparisons between the three study groups were checked with the Kruskal–Wallis test, followed by nonparametric post-hoc statistical tests for pairwise comparisons. The comparisons for repeated measurements (initial vs. day 5), were assessed with the Wilcoxon signed-rank test. A level of 0.05 was used for significance, and the two-tailed p-value was used for all statistical tests. All statistical calculations were computed in R software version 3.6.2. [R Core Team (2019). R: A language and environment for statistical computing. R Foundation for Statistical Computing. Vienna. Austria. http://www.R-project.org/]^35^.

## Data Availability

The datasets generated during and/or analyzed during the current study are available from the corresponding author on reasonable request.
